# A tribute to Marc Caron (1946–2022)

**DOI:** 10.1172/JCI163201

**Published:** 2022-08-01

**Authors:** Bryan Roth

**Affiliations:** Department of Pharmacology, UNC School of Medicine, Chapel Hill, North Carolina, USA.

Neuroscience lost an exceptional leader in molecular neurobiology when Marc G. Caron, PhD, passed away April 25, 2022, at the age of 75 (Figure 1). Marc’s contributions to our understanding of G protein–coupled receptors generally, and dopamine receptors in particular, span nearly 50 years. Marc’s major accomplishments include the identification of dopamine receptors as regulating prolactin release (1) and being G protein coupled (2); molecular cloning of several dopamine receptors and transporters (3–5); identification of arrestin as a major mediator of GPCR signaling (6); and, in systematic studies, demonstrating the roles of various dopamine receptors and transporters in vivo via the use of mice with genetically deleted receptors and transporters (7, 8). Together with his long-time collaborator Robert (Bob) Lefkowitz (who received the Nobel Prize in Chemistry in 2012), Marc also made seminal contributions to our current understanding of GPCRs in normal and abnormal physiology. Marc’s work was instrumental in clarifying the role of dopamine receptors in substance use disorders as well as neuropsychiatric conditions and therapeutics (9, 10). Moreover, his work on β-arrestin signaling of GPCRs has enormous clinical implications related to drug development, including serving as the basis for biased analgesic ligands with lower potential for patient misuse (7). Marc published more than 650 papers, and his work, collectively, received nearly 140,000 citations. Marc was a true giant in the field of GPCR research, and he will be missed greatly by the scientific community.

A native of Canada, Marc received his BSc in chemistry from Laval University and his PhD from the University of Miami. He was the first of Bob Lefkowitz’s amazing crop of postdoctoral fellows, and during this time Marc provided the initial insights into the biochemical mechanisms by which GPCRs couple to their downstream effectors. He was subsequently recruited to Laval in 1975 and then in 1977 returned to the United States as a tenure-track assistant professor at Duke University, where he spent the rest of his remarkable career. Marc was a Howard Hughes Investigator at Duke from 1992 to 2004 and was eventually promoted as the James B. Duke Professor — a position he held at his death. Marc received a number of awards and accolades, including election to the American Academy of Arts and Sciences and the Julius Axelrod Award in Pharmacology from the American Society for Pharmacology and Experimental Therapeutics.

I came to know Marc intimately during our tenure on the Editorial Board of the *Journal of Clinical Investigation* (2012–2017). As readers of the *JCI* may or may not be aware, the Editors met as a team once per week to discuss and make recommendations on all manuscript submitted to the *JCI*, and I had the pleasure to sit next to Marc all those years. Marc was one of those rare scientists who strove to see the potential “hidden gems” among the many dozens of papers he handled every month. His reviews and comments were invariably well reasoned and thorough, and he always emphasized the upside of the work being discussed (while some of us, myself included, tended to focus on the negatives). Even though the discussions occasionally were contentious, I never heard a disparaging remark from Marc regarding anyone’s contrary opinion or regarding the merits of the particular paper under discussion. One thing Marc emphasized frequently is that he felt that no experiment (assuming the appropriate controls were run) was flawed or incorrect; even in the case of work that was controversial, he would remind us that “there is very little in science that is truly irreproducible” (in his characteristic French-Canadian accent). Marc went out of his way to give the authors the benefit of revision and made sure that his recommendations for additional experiments were not excessively burdensome.

Marc also was a frequent reviewer for the NIH, and although I never saw him in action, I have the suspicion that he was the lone positive reviewer on several of my grants. In the early 1990s, I remember struggling to obtain my very first NIH grant, which received what is now a “triage-able” score on the first submission and a somewhat better score on the second. Buried in the reviews of the second submission was a suggestion to focus on particular aspects of GPCR pharmacology that were in Marc’s area of expertise (regulation and structure-activity studies). I did so and on the third and final review received a fundable score. My sense is that it was likely Marc who advocated for my work, and when I asked him about this many years later, he said, in effect, “Oh so you finally got that grant?” I told him many times over the years that I felt I owed my entire scientific career to him — not the least because of his advocacy, but also for his seminal work, which served as a springboard and continues to inspire my lab and hundreds of others worldwide.

Marc was also an exceptional mentor and trained a legion of accomplished scientists. Ten years ago, on the occasion of his 65th birthday, Duke held a reunion of his many postdocs and collaborators. The room was filled to capacity, and I was astounded at the number and quality of his former trainees and their work. Marc was also exceptionally dedicated to his family. He was preceded in death by his wife, Réjeanne Caron, and is survived by his children Kathleen Caron (with whom I also had the pleasure to work on the *JCI* Editorial Board), Melissa Caron Grahn, and Nelson Caron. He was a devoted grandfather to his six grandchildren.

Marc was a prolific scientist who was actively working on many exciting projects at the time of his death. He is greatly missed by myself, the GPCR community, and his legions of friends and colleagues.

## Figures and Tables

**Figure 1:**
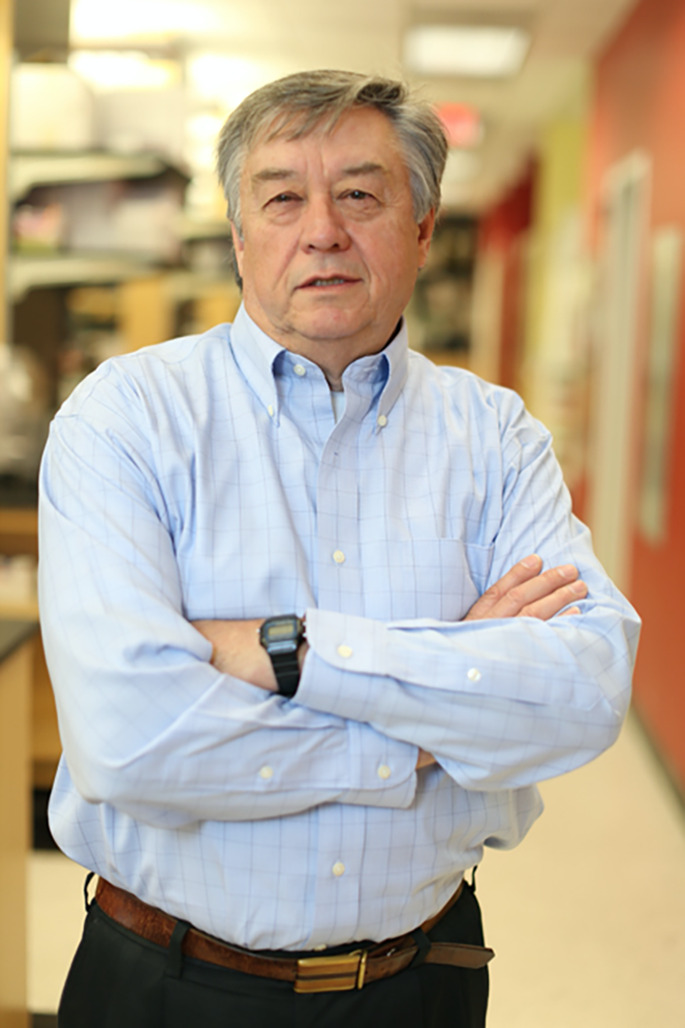
Marc Caron, PhD.

## Version 1 08/01/2022

Electronic publication
